# Primary intraosseous Rosai–Dorfman disease: An analysis of clinicopathologic characteristics, molecular genetics, and prognostic features

**DOI:** 10.3389/fonc.2022.950114

**Published:** 2022-09-15

**Authors:** Xin Weng, Yajie Yang, Meng Zhang, Chang Cai, Yanhua Sun, Xikang Wu, Rongrong Zhang, Huihui Gui, Wei Li, Qizhong Xu, Xia Liu

**Affiliations:** ^1^ Department of Pathology, Shenzhen Second People’s Hospital, Shenzhen University First Affiliated Hospital, Shenzhen, China; ^2^ Department of Joint and Musculoskeletal Tumor, Shenzhen Second People’s Hospital, Shenzhen University First Affiliated Hospital, Shenzhen, China; ^3^ Department of Radiology, Shenzhen Second People’s Hospital, Shenzhen University First Affiliated Hospital, Shenzhen, China

**Keywords:** primary, bone, Rosai–Dorfman disease, clinicopathologic features, OCT2, cyclin D1, progression-free survival (PFS), MAPK pathway

## Abstract

**Background:**

Rosai–Dorfman disease (RDD) is a rare histiocytic proliferative disorder of uncertain pathogenesis. Most patients present with proliferation in the lymph nodes manifesting as adenopathy; however, RDD may primarily arise in a variety of extranodal sites, including the bone, which is a great challenge in the diagnosis. The clinicopathological characteristics and prognostic features of primary intraosseous RDD have not been well characterized.

**Methods:**

We retrospectively analyzed the clinicopathologic and prognostic features of four cases of primary intraosseous RDD during the past 10 years in our hospital, with a review of an additional 62 cases with complete follow-up data from the literature.

**Results:**

Primary intraosseous RDD was identified in 0.14% (4/2,800) of total bone biopsies performed at our institution over the study period. According to our retrospective analysis, a total of 18 cases of primary lymph node, skin, or other non-osseous site-based RDD were diagnosed in our hospital. The ages of the 66 total patients ranged from 1.5 to 76 years, with a median age of 25 years. There were 31 male and 35 female patients, with a male-to-female ratio of 0.89:1. Primary intraosseous RDD occurred most often in the bones of the extremities (60.6%, 40/66), with the proximal tibia being the most common location; 39.4% (26/66) of the cases arose in the axial skeleton, predominantly in the vertebra and craniofacial bones. Solitary masses and multiple tumors were present in 84.8% (56/66) and 15.2% (10/66) of the cases, respectively. Pain of the affected area was the most common presenting symptom. Radiographically, the lesions were lytic with well-defined and usually sclerotic margins. Immunohistochemistry showed that large histiocytes from patients with RDD were positive for OCT2, in addition to S100 and CD68. Molecular tests were performed in seven reported cases and four of our cases. All the 11 cases were non-decalcified. PCR results showed that there were no *BRAF-V600E*, *KRAS*, or *NRAS* mutations in primary intraosseous RDD; only one case with both RDD and Langerhans cell histiocytosis showed *BRAF-V600E* mutation. The survival data showed that 22.7% (15/66) of the patients experienced recurrences or developed RDD at distant sites during the follow-up period (median follow-up, 13 months; range, 1–106 months). The 5-year progression-free survival (PFS) of the patients with primary intraosseous RDD was 57.5%. We found that there was a significant difference in PFS between female and male patients (*p* = 0.031). However, there was no statistically significant difference in PFS between patients with solitary masses and multiple tumors (*p *= 0.698). Similarly, no statistically significant differences in PFS were found between the different age groups (*p *= 0.908) or tumor locations (*p *= 0.728).

**Conclusion:**

Primary intraosseous RDD is an extremely rare disease. The diagnosis of RDD may be quite challenging because of its non-specific clinical presentation and imaging. Immunohistochemistry showed that large histiocytes were positive for OCT2 in addition to S100 and CD68, which may be helpful for differential diagnosis. Molecular detection showed that RDD may be related to the MAPK pathway, though these results are also ultimately not specific. The pathogenesis of RDD is yet to be elucidated, but recent studies suggest possible clonality of hyperproliferative histiocytes.

## 1 Introduction

Rosai–Dorfman disease (RDD), as a synonym for sinus histiocytosis with massive lymphadenopathy, was first described in 1965 (1), although it was not recognized as a distinct clinical entity until 1969 (2). RDD is a histiocytic proliferation disorder characterized by large S100-positive histiocytes exhibiting emperipolesis (3). RDD usually involves the lymph nodes, most frequently in the neck. As a result, patients typically present with painless bilateral cervical lymphadenopathy, in conjunction with fever, leukocytosis, and elevated erythrocyte sedimentation rate (4). Extranodal disease may occur as a primary process or in association with bone involvement.

Primary intraosseous RDD is an extremely rare disease. To date, only about 100 cases of intraosseous RDD have been reported, mainly as case reports rather than as study series. Because of its rarity, the clinicopathological characteristics and prognostic features of primary intraosseous RDD have not been well described. In an attempt to expand the known clinicopathologic and molecular genetic characteristics, as well as prognostic features, we retrospectively analyzed four cases of primary intraosseous RDD in our hospital with a review of an additional 62 cases with complete follow-up data from the literature.

## 2 Materials and methods

### 2.1 Case selection

All cases of primary intraosseous RDD diagnosed from January 2012 to July 2022 in the Department of Pathology, Shenzhen Second People’s Hospital, Shenzhen University First Affiliated Hospital, Shenzhen, Guangdong, China, were retrospectively analyzed. Primary intraosseous RDD cases were collected. The inclusion criteria of this retrospective study were as follows: 1) imaging showed no cervical mass or systemic superficial lymph node enlargement and 2) intraosseous lesion as the initial presentation without extraskeletal or lymph node manifestations at presentation. Patients with RDD with evidence of systemic disease or associated lymphadenopathy were excluded. The clinical data collected for analysis included age, gender, location, clinical presentation, imaging, treatment regimens, and survival data. All patients provided written informed consent for the collection and publication of their medical information during their first visit to the hospital.

We also performed an extensive literature search for reported cases of primary intraosseous RDD in PubMed (www.ncbi.nlm.nih.gov/pubmed/) using different combinations of keywords in the title/abstract field, including “primary”, “bone”, “intraosseous”, “sinus histiocytosis with massive lymphadenopathy”, “RDD”, and “Rosai–Dorfman”. Cases in English-language literature were carefully reviewed to extract essential clinicopathologic and prognostic data and to combine the cases that were repeatedly studied in different papers. A total of 62 cases of primary intraosseous RDD were retrieved from the literature and included in our review.

### 2.2 Immunohistochemical staining and *in-situ* hybridization

The specimens of these four cases of primary intraosseous RDD cases were formalin-fixed and paraffin-embedded and then sectioned at 4.0 μm thickness. The sections were stained using hematoxylin and eosin staining or were used for immunohistochemical examination. The immunohistochemical stains were performed on a Leica BOND-III Fully Automated IHC & ISH Staining System (Leica Biosystems Newcastle Ltd., England) with Bond Polymer Refine Detection Kit (Leica Biosystems; Catalog no. DS9800). Appropriate negative and positive controls were performed with satisfactory staining. The pretreatment methods, primary antibodies, and their working dilutions are listed in **Table S1**.

The EBV Probe *In Situ* Hybridization Kit (Zhongshan Golden Bridge Biotechnology Co. Ltd., Beijing, China; Catalog no. ISH-7001) was used to detect Epstein–Barr virus-encoded small RNAs according to the manufacturer’s protocol. The positive signals were a brownish-yellow color localized in the nuclei.

### 2.3 Molecular assays for gene mutations

*BRAF-V600E*, *KRAS*, and *NRAS* mutations were detected in the undecalcified and formalin-fixed paraffin-embedded samples using real-time PCR. Genomic DNA was extracted from tumor cell samples *via* the commercial AmoyDx^®^ FFPE DNA Kit (Amoy Diagnostic Co. Ltd., Xiamen, China; Catalog no. 8.02.23501X036G) according to the kit’s instructions. DNA (15 ng) was then examined for *BRAF-V600E*, *KRAS*, and *NRAS* mutations using commercial kits with a detection sensitivity of 1% mutation load (Human BRAF Gene V600E Mutation Fluorescence PCR Diagnostic Kit, Amoy, Catalog no. 8.0120301X024A; AmoyDx^®^ KRAS/NRAS Mutations Detection Kit, Amoy, Catalog no. 8.01.25402W006A) in an ABI 7500 real-time PCR machine (Applied Biosystems, CA, USA). FAM signals from the mutation detection system indicated the mutation status of the sample.

### 2.4 Statistical analysis

Progression-free survival (PFS) was defined as the years from the first diagnosis of primary intraosseous RDD to local recurrence, secondary lesions in other locations, or last follow-up. Those without evidence of the disease at last follow-up were treated as censored. The Kaplan–Meier method was used to estimate overall distributions, and the log-rank test was used to compare survival distributions between patient groups. *p*-values <0.05 (two-sided) were considered to be statistically significant. The life table method was used to estimate overall distributions. SPSS software (version 26.0 for Mac; SPSS Statistics Inc., IL, USA) was used for the analysis.

## 3 Results

### 3.1 Patients’ clinical characteristics

The major clinical features of the four cases of primary intraosseous RDD are summarized in **Table 1**. There were one female and three male patients, with ages ranging from 25 to 35 years. Primary intraosseous RDD was identified in 0.14% (4/2,800) of total bone biopsies performed at our institution over the study period. After retrospective analysis, a total of 18 cases of primary lymph node, skin, or other non-osseous site-based RDD in our hospital were diagnosed. All patients were Chinese without a history of RDD, and imaging showed no cervical mass or systemic superficial lymph node enlargement. The lesions arose in the humerus, skull, and vertebra, respectively. Clinically, patient #1 complained of pain in the left shoulder, with limited activity for more than 1 month. Patient #2 found a subcutaneous mass in his right forehead for 6 months. Patient #3 presented with limb numbness for 2 months when visiting the hospital. Patient #4 complained of left middle finger pain for 2 months.

**Table 1:** Clinical features of the four cases of primary intraosseous RDD in our hospital.

Case	Age (years)	Gender	Location	Clinical presentation	Imaging	Lesion	Treatment	Outcome (mo)	
#1	25	M	Left proximal humerus metaphysis, extended into the adjacent epiphysis	Left shoulder pain with limited activity for more than 1 month	Irregular cystic transparent area with focal sclerotic margins	S	Lesion curettage	NED, 12
#2	28	F	Right frontal bone	Subcutaneous mass of right forehead was found for 6 months	Bone defect area, soft tissue mass	S	Lesion excised	NED, 65
#3	32	M	Spinous process of C2–C5	Limbs with numbness for 2 months	MRI showed an enhancing intramedullary mass	S	Lesion excised	NED, 10
#4	35	M	Middle phalanx of the left middle finger	Left middle finger pain for 2 months	Irregular cystic transparent area with trabecular destruction and absorption	S	Lesion excised	NED, 2

F, female; M, male; mo, months; M, multiple; MRI, magnetic resonance imaging; NED, no evidence of disease; S, single.

After an extensive search of the English literature, we found 62 cases of primary intraosseous RDD with complete follow-up data (5–42). Some cases were included in two articles with different study purposes, and the data from these cases were carefully extracted and combined. The clinicopathologic features of these cases are summarized in **Table S2**. The brief clinicopathologic characteristics of the cases from the literature and our institution are summarized in **Table 2**.

**Table 2:** Summary of the brief clinicopathologic features of primary intraosseous RDD in the present study and the literature.

Characteristics	Present study	Literature	Total
Total cases	4	62	66
Male/female	3/1	28/34	31/35
Median age (range) (years)	/(25–35)	23 (1.5–76)	25 (1.5–76)
Location (extremital bone/axial skeleton)	2/2	38/24	40/26
Lesions (single/multiple)	4/0	52/10	56/10
*BRAF-V600E* mutation (positive/negative/unknown)	0/4/0	1/6/55	1/10/55
*KRAS* mutation (positive/negative/unknown)	0/4/0	0/7/55	0/11/55
*NRAS* mutation (positive/negative/unknown)	0/4/0	0/0/62	0/4/62
Median follow-up (mo)	/(*n* = 4)	14 (*n* = 62)	13 (*n* = 66)
5-year PFS	/	/	57.5%
Outcome (recurrence or progression/NED)	0/4	15/47	16/50

mo, months; NED, no evidence of disease; PFS, progression-free survival.

The ages of the 66 total patients ranged from 1.5 to 76 years, with a median age of 25 years. There were 31 male and 35 female patients, with a male-to-female ratio of 0.89:1. Primary intraosseous RDD occurred most often in the bones of the extremities (60.6%, 40/66), with the proximal tibia being the most common location; 39.4% (26/66) of the cases arose in the axial skeleton, predominantly in the vertebra and craniofacial bones. A solitary mass and multiple tumors were present in 84.8% (56/66) and 15.2% (10/66) of the cases, respectively. Pain of the affected area was the most common presenting symptom.

### 3.2 Radiology findings

**Figure 1:**
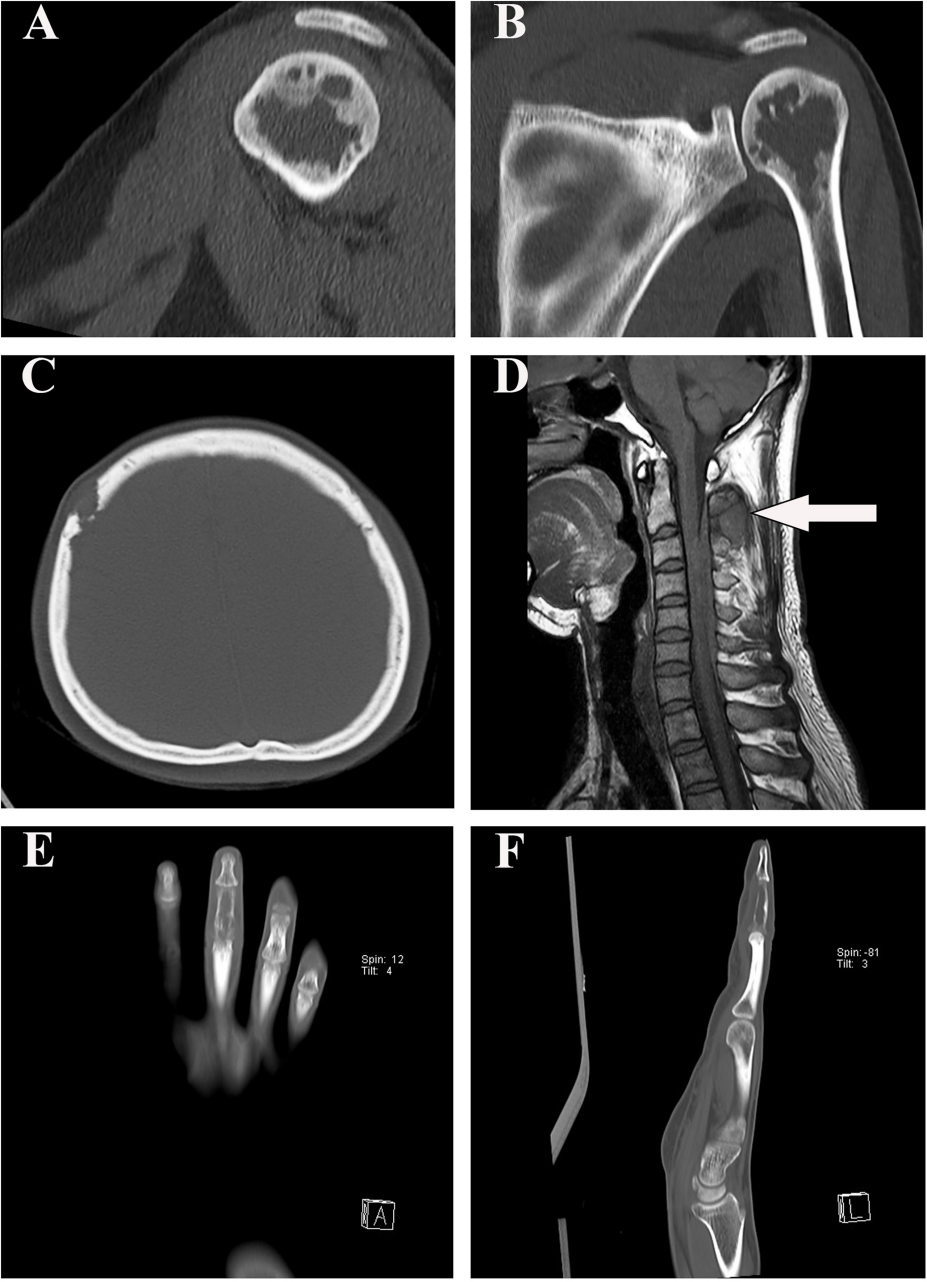
Radiographic findings in our four patients. **(A, B)** The sagittal and coronal computed tomography scan demonstrated that patient #1 had an irregular cystic transparent area in the left proximal humerus metaphysis, extended into the adjacent epiphysis. **(C)** Computed tomography image showed that the right frontal bone of patient #2 was damaged locally. **(D)** T1-weighted MRI showed that patient #3 had a hypointense epidural lesion in the spinous process of C2–C5 (arrow). **(E, F)** Patient #4 showed an irregular cystic transparent area with trabecular destruction and absorption in the coronal and sagittal computed tomography scan.

Imaging was available for the four patients. Patient #1 had an irregular cystic transparent area in the left proximal humerus metaphysis which extended into the adjacent epiphysis, with focal sclerotic margins (**Figures 1A, B**). The right frontal bone of patient #2 was damaged locally, with the inner and outer plates becoming thinner and extending into the soft tissue locally(**Figures 1C**). Patient #3 had an enhanced intramedullary mass in the spinous process of C2–C5, extending into the adjacent soft tissue and epidural space and causing compression of the spinal cord (**Figures 1D**). Patient #4 showed an irregular cystic transparent area with trabecular destruction and absorption (**Figures 1E, F**).

### 3.3 Pathology findings

#### 3.3.1 Histology

**Figure 2:**
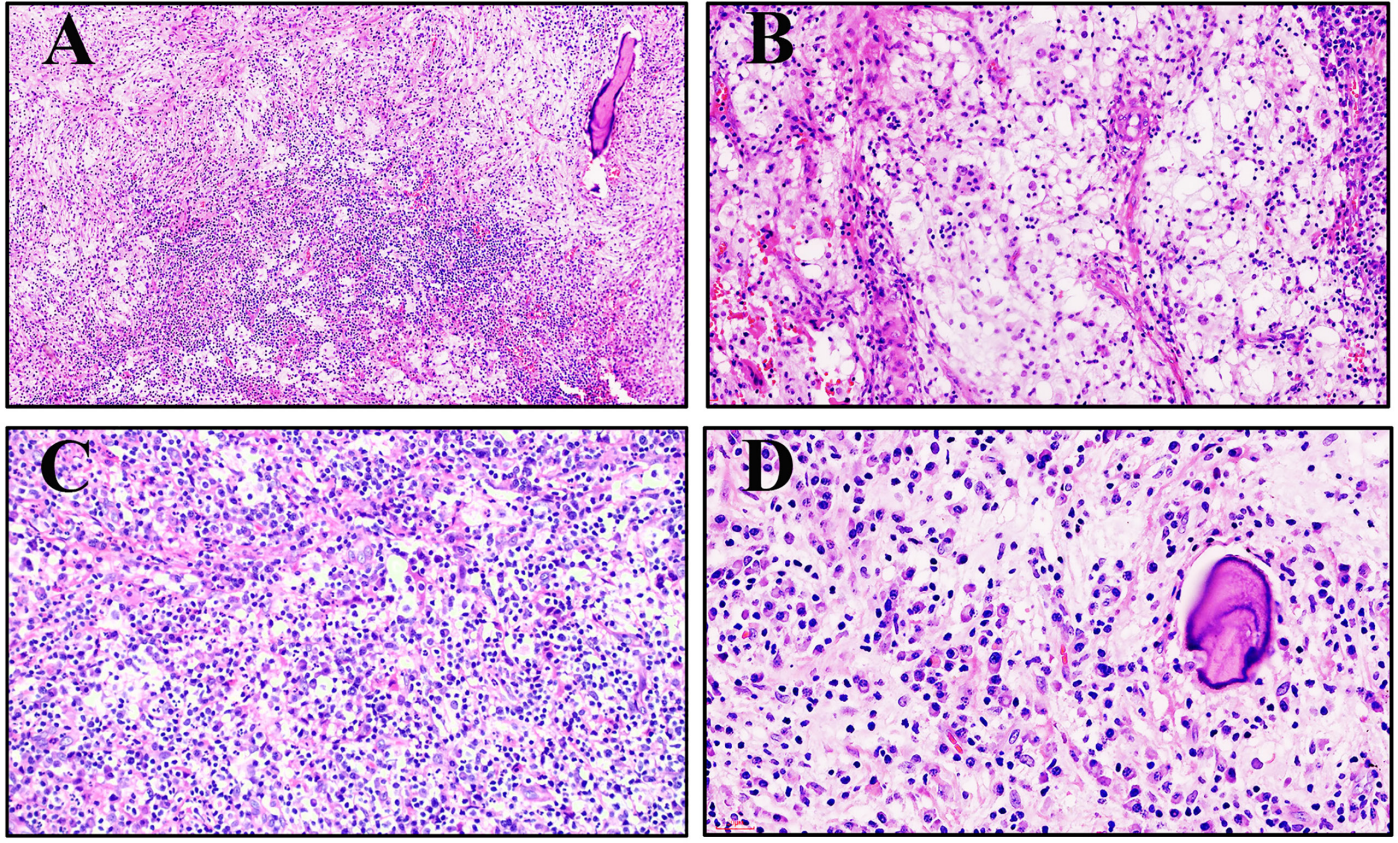
Morphology of primary intraosseous Rosai–Dorfman disease (RDD). **(A)** An infiltrative pattern of RDD in the medullary cavity (patient #3, hematoxylin and eosin ×100). **(B)** Large histiocytes with abundant clear to eosinophilic cytoplasm tended to form loose clusters surrounded by a mixed inflammatory infiltrate (patient #2, hematoxylin and eosin ×200). **(C)** Large histiocytes demonstrated emperipolesis of the neutrophils, lymphocytes, and plasma cells (patient #4, hematoxylin and eosin ×200). **(D)** The tumor cells are enmeshed in a fibrotic stroma that contains a great quantity of intermixed lymphocytes and plasma cells (patient #1, hematoxylin and eosin ×400).

The histomorphologic features of RDD in the bone and lymph nodes are not exactly the same. Classically, nodal RDD shows prominent sinusoidal involvement, but primary intraosseous RDD is poorly defined, replaces the marrow, infiltrates Haversian systems, and is associated with local bone resorption (**Figure 2A**). The mass is characterized by sheets and clusters of large histiocytes, with nuclei that range from round or oval to reniform, with fine or vesicular chromatin and prominent eosinophilic nucleoli (**Figure 2B**). The cytoplasm is abundant and pale eosinophilic, with conspicuous emperipolesis of the lymphocytes (lymphocytophagocytosis), plasma cells, or neutrophils (**Figure 2C**). The tumor cells were enmeshed in a fibrotic stroma that contained a great quantity of intermixed lymphocytes and plasma cells in patient #1 (**Figure 2D**).

#### 3.3.2 Immunophenotype

**Figure 3:**
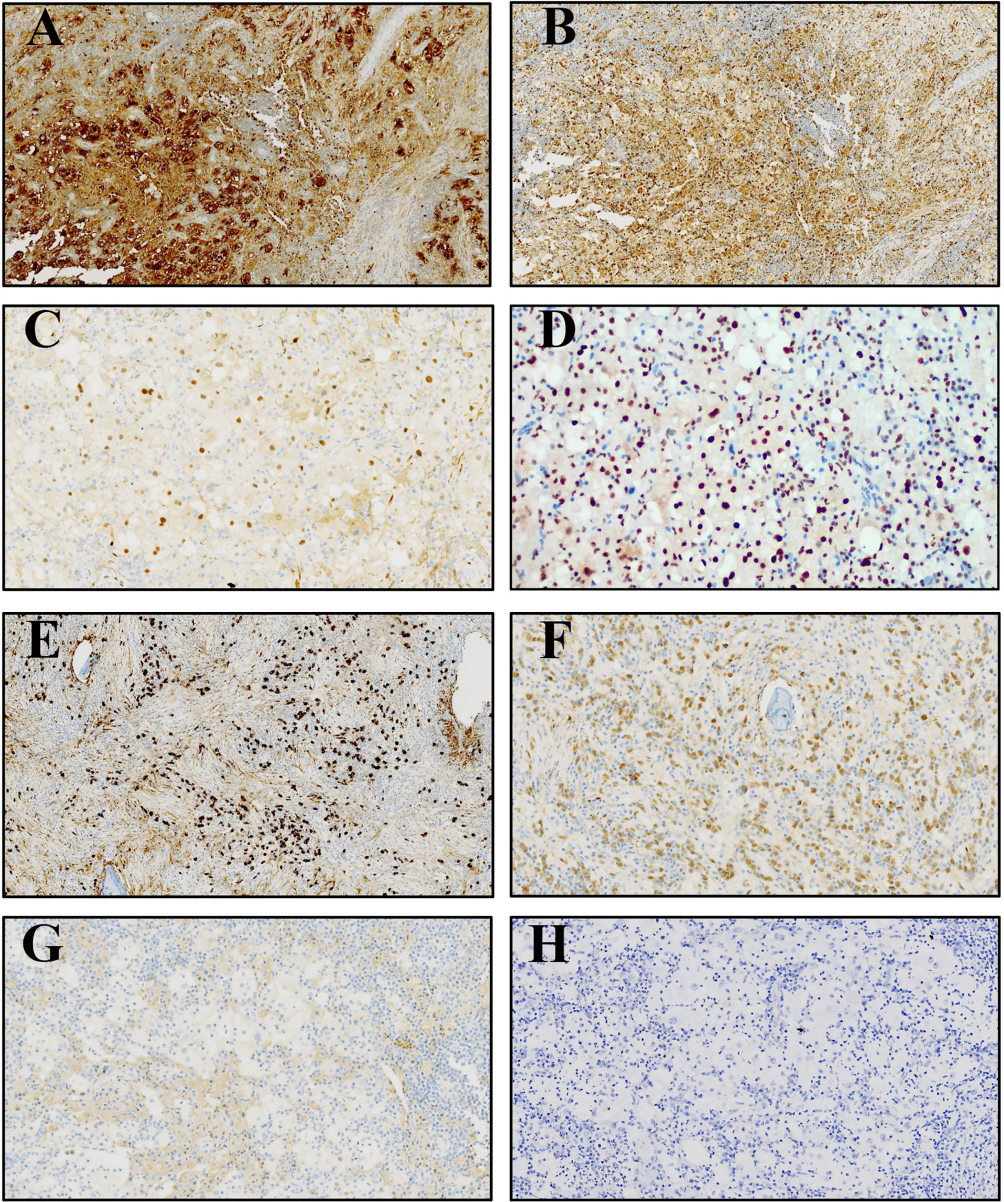
Immunophenotype of primary intraosseous RDD. **(A, B)** The large histiocytes were strongly positive for S100 and CD68, respectively. **(C)** The nuclear immunoreactivity for cyclin D1 was observed. **(D)** All cases were positive for OCT2. **(E, F)** The biopsy specimens of patient 1# had a large quantity of IgG4-positive and IgG-positive plasma cells. **(G, H)** Langerin and EBER were negative.

The results of immunohistochemistry are summarized in **Table 3**. All four cases had large histiocytes that were strongly positive for S100 and CD68 (**Figures 3A, B**). Nuclear immunoreactivity for cyclin D1 and OCT2 was observed in these cases (**Figures 3C, D**). Only one of the four cases showed CD163 positivity, and the other three were negative. All cases were negative for langerin (**Figure 3G**), CD1a, and EBER (**Figure 3H**). The biopsy specimens of patient #1 had more than 100 IgG4-positive plasma cells per high-power field and an IgG4/IgG ratio of more than 0.4:1 (**Figures 3E, F**). However, serum IgG4 (1.82 g/L, reference range: 0.03–2.01 g/L) and IgG (15.96 g/L, reference range: 7–16 g/L) levels were normal.

**Table 3:** Immunophenotype and EBV infection status of four primary intraosseous RDD.

Case	S100	OCT2	Cyclin D1	CD68	CD163	Langerin	CD1a	IgG4/IgG	EBER
#1	+	+	+	+	−	−	−	>40%	−
#2	+	+	+	+	−	−	−	/	−
#3	+	+	+	+	−	−	−	<40%	−
#4	+	+	+	+	+	−	−	<40%	−

+, positive; –, negative.

#### 3.3.3 Molecular pathology

PCR assays for *BRAF-V600E*, *KRAS*, and *NRAS* gene mutations were performed on the four cases. All four cases showed no mutations in *BRAF-V600E*, *KRAS*, or *NRAS*. However, due to limited conditions in our department, a larger next-generation sequencing (NGS) panel was not performed on these four cases, to assess for the presence of mutations in other genes within the MAPK pathway.

#### 3.3.4 Treatment and outcome

All four patients underwent surgical resections and did not accept further treatment. These four patients were followed up successfully until 18 April 2022. The follow-up interval ranged from 2 to 65 months. All patients survived without disease during the follow-up period.

**Figure 4:**
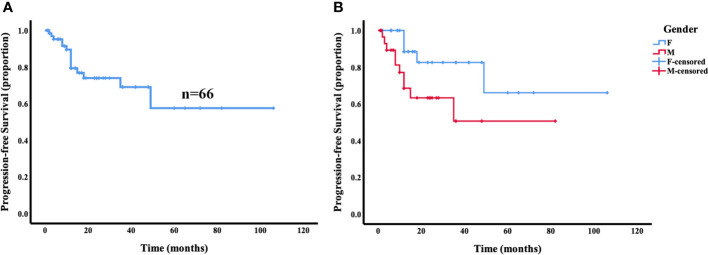
Progression-free survival (PFS) analysis for primary intraosseous RDD. The 5-year PFS of the patients with primary intraosseous RDD was 57.5% **(A)**. Female patients showed a trend toward superior progression-free survival compared with male patients (*p* = 0.031) **(B)**.

Of the 66 patients with survival data from our present study and reported in the literature, local recurrence and secondary lesions in other locations occurred in 22.7% (15/66) of the patients during the follow-up period (median follow-up, 13 months; range, 1–106 months). The 5-year PFS of the patients with primary intraosseous RDD was 57.5% (**Figure 4A**). Of note, the male patients had significantly lower 5-year PFS (50.5%) than the female patients (66.0%; *p* = 0.031) (**Figure 4B**). However, there was no statistically significant difference in PFS between patients with solitary masses and multiple tumors (*p* = 0.698). Similarly, no statistically significant differences were found in PFS between age groups (*p* = 0.908) and different tumor locations (*p* = 0.728).

## 4 Discussion

RDD is a rare histiocytic proliferative disorder of uncertain pathogenesis, which was first described in 1965 (1) and recognized as a distinct clinical entity in 1969 by Rosai and Dorfman (2). In the fifth edition of the WHO classification of soft tissue and bone tumors, RDD was classified as a hematopoietic neoplasm of the bone (3). Most of the patients present with lymph node involvement manifesting as adenopathy. In the present retrospective study, a total of 18 cases of primary lymph node, skin, or other non-osseous site-based RDD were diagnosed in our hospital. Extranodal disease may occur as a primary process or in association with nodal involvement (5). However, primary RDD of the bone is extremely rare and the occurrence of this entity remains unknown. Primary intraosseous RDD was identified in 0.14% (4/2,800) of total bone biopsies at our institution over the study period.

Due to its rarity, the clinicopathologic features and prognosis of primary intraosseous RDD have not been well characterized. To date, no known risk factors have been identified. After an extensive search of the English literature, only 62 cases of primary intraosseous RDD with complete survival data were identified. The median age of the patients was 24 years old, and there was no significant difference in age between genders. Pain of the affected area was the most common presenting symptom. Interestingly, we found that primary intraosseous RDD most occurred in the bones of the extremities, with the proximal tibia being the most common location. In addition, most of the tumors were single osteolytic lesions.

The etiology of RDD remains uncertain. The proposed mechanisms include immune dysfunction and possible viral infection. A few studies have reported that RDD may be related to the Epstein–Barr virus, human herpesvirus infections, and so on (43, 44). However, no Epstein–Barr virus or human herpesvirus infections were observed in primary intraosseous RDD from our present study and those reported in the literature, so any potential correlation between viral infection and the pathogenesis of primary intraosseous RDD cannot be clarified.

RDD has always been considered as a disease of histiocytic polyclonal hyperplasia, but in recent years, molecular genetics suggests that it may have a potential monoclonal pathogenesis. Fatobene and Haroche reported one case of nodal RDD with confirmed *BRAF-V600E* mutation, representing a promising therapeutic target, especially for patients with refractory or extensive disease (45). In addition, mutually exclusive *KRAS* and *MAP2K1* mutations were described in one-third of the cases of RDD, suggesting that this subgroup is clonal and involves activation of the MAPK/ERK pathway (46). Wu et al. reported an *NRAS* mutation in cutaneous RDD (47), indicating that *NRAS* mutations in the MAPK/ERK pathway may be involved in the pathogenesis of cutaneous RDD. Nevertheless, there was only one report on the molecular genetics of primary intraosseous RDD. Dong et al. detected *BRAF-V600E* and *KRAS* mutations in seven undecalcified primary intraosseous RDD cases, which showed that only one case with the concurrence of RDD and Langerhans cell histiocytosis (LCH) had *BRAF-V600E* mutation, suggesting that the *BRAF-V600E* mutation may be caused by LCH lesions rather than RDD lesions. Similarly, no *BRAF-V600E*, *KRAS*, and *NRAS* mutations were detected in our cases and all samples were not decalcified. Unfortunately, a larger NGS panel was not performed on our cases and the reported cases to assess for the presence of mutations in other genes within the MAPK pathway. Like our results, some studies (25, 48) have demonstrated immunohistochemical cyclin D1 expression in RDD cases including bone lesions, reflecting constitutive MAPK pathway activation in the pathogenesis of RDD. However, since few studies have been reported and no larger NGS panel has been performed, it is still unclear whether the primary intraosseous RDD is related to the activation of the MAPK pathway. Therefore, not only more cases but also more robust mutational analysis are needed to further confirm the findings.

Tracht et al. demonstrated a possible histologic overlap between RDD and the more common IgG4-related disease (IRD), which could cause problems in pathologic diagnosis (49). Another study demonstrated that RDD of the breast can show a significant increase in IgG4+ plasma cells as well as fibrosis, which may further complicate matters (50). In our present study, one case showed increased IgG4-positive plasma cells and IgG4/IgG ratio, but serum IgG4 and IgG levels were normal. Therefore, we speculate that RDD histomorphology may be associated with that of IRD, but a relationship between RDD and IRD has not been definitively established. A diagnosis of concurrence of RDD and IRD should integrate the pathological features, the number of IgG4-positive plasma cells, clinical manifestations, serological examinations. and so on.

The imaging characteristics of primary intraosseous RDD are not specific and often misleading. Radiographically, bone lesions are often misdiagnosed as osteomyelitis or LCH. Other entities in differential diagnosis include Erdheim–Chester disease, lymphoma, plasma cell myeloma, and metastatic disease (5). Osteomyelitis is a necrotizing and sclerosing bone disease dominated by inflammation, often including numerous neutrophils and with frequent periosteal reactive bone formation. Histologically, the mixed inflammatory infiltrate with focal neutrophilic micro-abscesses, occasional multinucleated giant cells, and granuloma-like histiocyte collections may suggest infection or granulomatous disease. However, the characteristic S100-positive histiocytes with emperipolesis are not seen in either condition. As the name suggests, LCH is dominated by the proliferation of Langerhans histiocytes. Langerhans histiocytes are usually found in granuloma-like clusters and have characteristic elongated, indented, grooved, or convoluted nuclei with inconspicuous nucleoli. In addition to the S100 protein, they are consistently positive for CD1a and langerin, which are not expressed by RDD histiocytes. Erdheim–Chester disease is a multisystemic proliferative histiocytic disorder, characterized by long bone involvement with bilateral and symmetrical sclerotic lesions. Frequently, there is extraskeletal involvement including the cardiovascular system, central nervous system, kidneys, and lungs. Histologically, there is a proliferation of foamy histiocytes within the marrow spaces with associated fibrosis and thickening of bone trabeculae. The proliferating histiocytes are positive for CD163 and CD68 and are usually negative for S100. They are also negative for CD1a and langerin. Emperipolesis is not seen in Erdheim–Chester disease. Metastatic carcinoma and melanoma can be ruled out by histomorphology and the lack of expression of epithelial and melanocytic markers.

Surgical resection or curettage is the most common treatment of primary intraosseous RDD. At present, there is great controversy about the relative benefits of postoperative adjuvant radiotherapy and steroids therapy (51). The prognosis is good, but local recurrences or secondary lesions in other locations may occur after surgery in some cases. Our analysis showed that the 5-year PFS of patients with primary intraosseous RDD was 57.5%. Interestingly, our analysis of all prior reported cases showed that female patients had a trend toward superior PFS compared with male patients. No statistically significant difference was found in PFS between patients with different age groups, tumor locations, or number of lesions.

### 4.1 Conclusions

Primary intraosseous RDD is an extremely rare disease. Diagnosis of the disease may be quite challenging because of its variable clinical manifestations, non-specific imaging findings, and background mixed with inflammatory infiltrate. Immunohistochemistry showed that large histiocytes from patients with RDD were positive for OCT2 in addition to S100 and CD68 and negative for CD163, which may be helpful for differential diagnosis. Molecular detection showed that RDD may be related to the MAPK pathway, though these findings are ultimately not specific. The pathogenesis of RDD is yet to be elucidated, but recent studies suggest possible clonality.

## Data availability statement

The original contributions presented in the study are included in the article/**Supplementary Material**. Further inquiries can be directed to the corresponding author.

## Ethics statement

All patients provided written informed consent for the collection and publication of their medical information during the first visit to the hospital.

## Author contributions

XW designed the study and wrote the manuscript. YJY and MZ contributed to the clinical collection, as well as data analysis. CC and YHS provided the cases. XKW performed molecular detection. RRZ and HHG performed immunohistochemical staining and *in-situ* hybridization (ISH). WL collected the follow-up information. QZX offered imaging data. XL provided supervision, conceived the project, and provided leadership. All authors contributed to the article and approved the submitted version.

## Conflict of interest

The authors declare that the research was conducted in the absence of any commercial or financial relationships that could be construed as a potential conflict of interest.

## Publisher’s note

All claims expressed in this article are solely those of the authors and do not necessarily represent those of their affiliated organizations, or those of the publisher, the editors and the reviewers. Any product that may be evaluated in this article, or claim that may be made by its manufacturer, is not guaranteed or endorsed by the publisher.

## Abbreviations

IRD, IgG4-related disease; LCH, Langerhans cell histiocytosis; PFS, progression-free survival; RDD, Rosai–Dorfman disease.
