# Supplementary material for: Primary intraosseous Rosai–Dorfman disease: An analysis of clinicopathologic characteristics, molecular genetics, and prognostic features
Source: Front Oncol. 2022 Sep 15;12:950114. doi: 10.3389/fonc.2022.950114 (PMC9520307; doi:10.3389/fonc.2022.950114)
Supplement: Supplementary file 1 [file Table_1.docx]

**Table S1. Primary Antibodies and Conditions Used for Immunohistochemical Staining**

| **Antigen** | **Clone** | **Dilution** | **Antigen Retrieval** | **Manufacturer** |
| --- | --- | --- | --- | --- |
| S-100 | 4C4.9 | 1:150 | HP EDTA | Maixin Biotech |
| CyclinD1 | SA38-08 | Working solution | HP EDTA | Long Island Antibody |
| CD68 | KP1 | 1:100 | HP EDTA | Maixin Biotech |
| langerin | 12D6 | Working solution | HP EDTA | Maixin Biotech |
| CD1a | 010 | Working solution | HP EDTA | Maixin Biotech |
| IgG4 | MRQ-44 | Working solution | HP EDTA | Maixin Biotech |
| IgG | Polyclonal antibody | 1:1000 | HP EDTA | Maixin Biotech |
| OCT2 | MRQ-2 | Working solution | HP EDTA | Maixin Biotech |
| CD163 | 10D6 | Working solution | HP EDTA | Maixin Biotech |

HP EDTA: boiling with EDTA (1 mmol/L pH 9.0) under high pressure.
