# Supplementary material for: Primary intraosseous Rosai–Dorfman disease: An analysis of clinicopathologic characteristics, molecular genetics, and prognostic features
Source: Front Oncol. 2022 Sep 15;12:950114. doi: 10.3389/fonc.2022.950114 (PMC9520307; doi:10.3389/fonc.2022.950114)
Supplement: Supplementary file 2 [file Table_2.docx]

**Table S2. Clinical Features of the 62 Cases of Primary Intraosseous RDD Reported in the Literatures**

| **Case** | **Age (y)** | **Gender** | **Location** | **Clinical presentation** | **Imaging** | **Lesion** | | **Treatment** | **Outcome (mo)** |
| --- | --- | --- | --- | --- | --- | --- | --- | --- | --- |
| Demicco EG et al. (5) | 46 | F | Distal femur, humerus | Pain | Lytic, multiloculated | M | NA | | Progression, 18 |
| Demicco EG et al. (5) | 17 | M | Left proximal tibia | Pain | NA | S | NA | | Progression, 3 |
| Demicco EG et al. (5) | 44 | F | Right clavicle | NA | Lytic | S | NA | | NED, 16 |
| Demicco EG et al. (5) | 45 | F | Skull, frontal and parietal regions | Seizures, headache | Lytic, well defined | M | NA | | NED, 42 |
| Demicco EG et al. (5) | 19 | M | Tibia, proximal humeral metadiaphysis | Pain | Lytic, well defined, multiloculated | M | NA | | NED, 36 |
| Demicco EG et al. (5) | 18 | M | Left maxilla | NA | NA | S | NA | | Progression, 35 |
| Demicco EG et al. (5) | 3 | M | Left calcaneus | Pain | Lytic, well defined, unilocular | S | NA | | NED, 82 |
| Demicco EG et al. (5) | 22 | F | Left proximal tibial metaphysis | Tibial tenderness | Lytic, well-defined | S | NA | | NED, 106 |
| Demicco EG et al. (5) | 19 | F | Right sacrum | NA | Lytic | S | NA | | Progression, 12 |
| Demicco EG et al. (5) | NA | M | Right distal femur | NA | Lytic | S | NA | | Progression, 12 |
| Demicco EG et al. (5) | 11 | F | Left proximal tibia | NA | NA | S | NA | | NED, 12 |
| Demicco EG et al. (5) | 56 | M | Right fourth metacarpal | Pain | Lytic, expansile | S | NA | | Progression, 10 |
| Xu J et al. (6) | 56 | F | Right proximal tibia | Pain | Lytic | S | Lesion curettage | | NED, 48 |
| Vithran DTA et al. (7) | 8 | F | Right tibia | Pain | Round cystic bone defect area with sclerotic margins | S | Lesion excised | | NED, 6 |
| Tripathy K et al. (8) | 52 | F | Left scaphoid bone | Pain | Lytic | S | Lesion curettage | | NED, 9 |
| Tekin U et al. (9) | 23 | F | Right mandibular premolar area | Pain | Lytic | S | Lesion excised | | NED, 36 |
| Sundaram C et al. (10) | 60 | F | The distal femur and mid-fibula | Pain | Osteolytic lesions with foci of sclerosis and shaggy solid periosteal reaction | M | Lesion curettage | | NED, 30 |
| Shulman S et al. (11) | 6 | F | The left third rib and multiple pulmonary nodules | Cough, fever, soft tissue mass | Abnormal lesion | M | Corticosteroids | | NED, 72 |
| Shulman S et al. (11) | 8 | F | The distal femur, proximal tibia, and parietal skull bone | Pain | Lytic | M | Corticosteroids | | NED, 36 |
| Ross AB et al. (12) | 76 | F | Left radial diaphysis, skull and left clavicle | Pain | Lytic | M | Lesion curettage | | NED, 2 |
| Ross AB et al. (12) | 20 | M | The distal left radial metaphysis and epiphysis | Pain | A lucent lesion with numerous internal septations | S | Lesion curettage | | NED, 1 |
| Robert EG et al. (13) | 23 | F | Sacrum | left leg pain and foot dorsal and plantar flexion weakness | Lytic | S | Lesion excised | | NED, 12 |
| Paryani NN et al. (14) | 49 | F | The mid distal femoral bone | Pain | A lobulated focus | S | Lesion curettage | | Progression, 12 |
| Parekh A et al. (15) | 34 | F | The distal radial metaphysis | Pain | Lytic | S | Lesion excised | | NED, 6 |
| Miniello TG et al. (16) | 39 | F | The right maxilla | Teeth mobility and pain | Lytic | S | corticosteroids | | NED, 60 |
| Mansoori J et al. (17) | 39 | F | The medial malleolus as well as the lateral calcaneus | Pain | Lytic | M | Lesion excised | | NED, 6 |
| Izubuchi Y et al. (18) | 2 | M | The right ilium | Limping of the right lower extremity | Lytic | S | No | | NED, 18 |
| Hsu AR et al. (19) | 16 | M | The right scapula glenoid | Pain | Cystic lesion with a sclerotic rim | S | Lesion curettage | | NED, 1.5 |
| Goel MM et al. (20) | 7 | F | The shaft of the tibia | Pain | Lytic | S | NA | | NED, 6 |
| George J et al. (21) | 41 | F | The proximal to-mid diaphysis of the left radius | Pain | Lucent | S | Lesion excised | | NED, 14 |
| Baker JC et al. (22) | 19 | M | The distal left femur | Pain | Lytic | S | Lesion curettage | | NED, 23 |
| Rittner RE et al. (23) | 15 | M | Left femur and the left pelvic bones | Fever, shivering and pain | Lytic | M | Nonsteroidal antirheumatics | | NED, 6 |
| Okay E et al. (24) | 17 months | M | Left talus | Pain | NA | S | Lesion curettage | | NED, 12 |
| Dong RF et al. (25) | 27 | F | Left proximal femur | Pain | Well-circumscribed lytic lesion | S | Lesion curettage | | Progression, 49 |
| Dong RF et al. (25) | 18 | F | Right ischium | Pain | Lytic, soft tissue lesion | S | No | | NED, 42 |
| Dong RF et al. (25) | 23 | M | Left proximal tibia | Pain | Well-circumscribed lytic lesion | S | Lesion curettage | | NED, 27 |
| Dong RF et al. (25) | 2 | M | Left humeral metaphysis | Swelling and tenderness | Lytic, soft tissue lesion | S | Lesion curettage | | NED, 28 |
| Dong RF et al. (25) | 41 | M | Skull | Pain | Lytic | S | Lesion excised | | NED, 25 |
| Dong RF et al. (25) | 44 | F | Right humeral metaphysis | Pain | Lytic | S | Lesion excised | | NED, 23 |
| Dong RF et al. (25) | 35 | F | Right tibia | Pain | Well-circumscribed lytic lesion | S | No | | NED, 19 |
| Dong RF et al. (25) | 30 | F | Right tibia diaphysis | Pain | Lytic | S | Lesion excised | | NED, 9 |
| Dong RF et al. (25) | 23 | M | Right hand ring finger | Swelling and pain | Lytic | S | Lesion curettage | | Recurrence,12 |
| Dong RF et al. (25) | 26 | M | Left scapula | Pain | Well-circumscribed lytic lesion | S | Lesion curettage | | NED, 7 |
| Dong RF et al. (25) | 64 | M | Pedicle of T6, nasal cavity | Weakness of both lower limbs with numbness | Lytic, soft tissue lesion | M | Vertebral resection | | Progression, 15 |
| Dong RF et al. (25) | 38 | F | Vertebral bodies of L4 | Pain, numbness of left lower limb | Lytic | S | Vertebral resection | | NED, 25 |
| Kumar A et al. (26) | 35 | M | C6-T7 | Pain and unsteadiness of gait | T2-weighted MRI showed a hypo-intense epidural lesion | S | Lesion excised, Vinblastin | | NED, 24 |
| El Molla M et al. (27) | 76 | M | C2-C3 | Progressive right arm weakness and right footdrop | T1-weighted MRI showed an enhancing intramedullary mass | S | laminectomy | | NED, 12 |
| Nguyen PX et al. (28) | 19 | M | T6-T9 | Progressive bilateral leg weakness and back pain | MRI showed a heterogeneous hyperintense on T2W, and isointense on T1W | S | Lesion excised | | NED, 4 |
| Hamels J et al. (29) | 1.5 | M | Left distal radius | Pain/swelling | Lytic | S | Curettage | | NED, 36 |
| Lewin JR et al. (30) | 7 | M | Right fifth metacarpal | Pain/swelling | Expansile, lytic | S | Ray amputation | | NED, 24 |
| Nawroz IM et al. (31) | 11 | M | Right distal radius | Pain/swelling | Lytic | S | Curettage | | NED, 48 |
| Allegranza A et al. (32) | 14 | F | Right parietotemporal bone | NA | Lytic, ill defined | S | Curettage | | NED, 17 |
| Kademani D et al. (33) | 44 | F | Left maxilla | Pain/swelling | Lytic | S | Partial maxillectomy | | NED, 14 |
| Loh SY et al. (34) | 57 | F | Left triquetrum | Pain | Lytic | S | Curettage, RT | | NED, 12 |
| Mota Gamboa JD et al. (35) | 19 | F | Left proximal tibia | Pain/swelling | Lytic, ill defined | S | Resection | | NED, 10 |
| Rodriguez-Galindo C et al. (36) | 9 | F | Left frontal bone | Pain/swelling | Lytic, well defined | S | Curettage | | Recurrence,12 |
| Al-Saad K et al. (37) | 17 | M | T9 vertebra | Pain, loss of sensation, gait abnormality | Lytic | S | Resection | | Recurrence, 8 |
| Miyake M et al. (38) | 38 | F | Right femur | Pain | Lytic, poorly defined | S | Conservative | | NED, 6 |
| Tubbs RS et al. (39) | 13 | M | Right parietal and mastoid bone | Pain | Lytic | S | Mastoidecomy, CT | | Recurrence, 4 |
| Yoon AJ et al. (40) | 35 | M | Left distal fibula | Pain | Lytic, ill defined | S | Curettage | | Recurrence, 2 |
| Keskin A et al. (41) | 29 | F | Left maxilla | Pain/swelling | Lytic, ill defined | S | Resection | | NED, 16 |
| DeFelice DS et al. (42) | 46 | M | Right proximal tibia | Pain | Lytic | S | Right proximal tibia excision | | Progression, 8 |

CT, chemotherapy; F, female; M, male; mo, months; M, multiple; MRI, magnetic resonance imaging; NA, not available; NED, no evidence of disease; RT, radiotherapy; S, single.
